# Mechanisms, Pathophysiology and Current Immunomodulatory/Immunosuppressive Therapy of Non-Infectious and/or Immune-Mediated Choroiditis

**DOI:** 10.3390/ph15040398

**Published:** 2022-03-24

**Authors:** Ioannis Papasavvas, Ilknur Tugal-Tutkun, Carl P. Herbort

**Affiliations:** 1 Retinal and Inflammatory Eye Diseases, Centre for Ophthalmic Specialized Care (COS), Rue Charles-Monnard 6, CH-1003 Lausanne, Switzerland; i.s.papasavvas@gmail.com; 2 Department of Ophthalmology, Istanbul Faculty of Medicine, Istanbul University, Istanbul 34093, Turkey; tutkunilknur@gmail.com

**Keywords:** choriocapillaritis, stromal choroiditis, multimodal imaging, indocyanine green angiography (ICGA), immunosuppressive therapy, biological therapies

## Abstract

Non-infectious choroiditis comprises immune-mediated diseases resulting from diverse pathophysiological mechanisms. These conditions are sub-divided into two main groups, (1) diseases of the choriocapillaris and (2) diseases of the choroidal stroma. The purpose of this study is to expose the pathophysiology of the most common diseases of both these groups and recommend the optimal immunomodulatory/immunosuppressive therapy of each analyzed condition based on literature data and data from our own centers. Material and Methods: Narrative review. In the group of choriocapillaritis entities or primary inflammatory choriocapillaropathies (PICCPs) including multiple evanescent white dot syndrome (MEWDS), acute posterior multifocal placoid pigment epitheliopathy (APMPPE), idiopathic multifocal choroiditis (MFC) and serpiginous choroiditis (SC), as well as secondary choriocapillaritides including acute syphilitic posterior multifocal placoid chorioretinitis (ASPMPC) and tuberculosis-related SC (TB-SC), were analyzed. In the group of stromal choroidites, HLA-A29 birdshot retinochoroiditis (BRC) and Vogt-Koyanagi-Harada (VKH) disease were included. For each entity a literature search, in the PubMed database, on treatment was performed and analyzed and the therapeutic attitudes of our own centers were presented. Management of immune-mediated choroiditis implies vigorous immunosuppressive therapy given in a prompt and prolonged fashion in most of these entities.

## 1. Introduction

The management of uveitis in general and of non-infectious/immune-mediated choroiditis in particular is far from reaching a consensus among practitioners and is the object of diverse, if not divergent approaches [1,2,3]. Most of non-infectious choroiditis entities can be classified as severe uveitis conditions for which appropriate therapy is crucial in order to achieve disease control, disease remission or even disease cure [4].

Non-infectious/immune-mediated choroiditis is the result of at least two main pathophysiological mechanisms, (1) immune-mediated choriocapillaris non-perfusion produced by choriocapillaritis and (2) auto-immune infiltration of the choroidal stroma producing stromal choroiditis [5].

The group of choriocapillaritis diseases includes primary inflammatory choriocapillaropathies (PICCPs) [6] possibly triggered by unknown viral agents [7]. Some of these conditions may be self-resolving such as multiple evanescent white dot syndrome (MEWDS) [8], but most of them are sight-threatening if prompt and appropriate treatment is not applied; they include acute posterior multifocal placoid pigment epitheliopathy also called more appropriately acute multifocal ischemic choriocapillaritis (APMPPE/AMIC) [9], idiopathic multifocal choroiditis (MFC) [10] and serpiginous choroiditis (SC) [11]. Additionally, some choriocapillaritis entities result from immune-mediated mechanisms triggered by known infectious agents such as tuberculosis related SC (TB-SC) [12,13] and acute syphilitic posterior placoid chorioretinitis (ASPPC) [14]. For all these conditions the optimal therapy will be determined through a literature search and data generated from our own group.

In the group of stromal choroidites, Vogt-Koyanagi-Harada disease (VKH) [15] and HLA-A29 birdshot retinochoroiditis (BRC) [16] were analyzed in the same fashion.

All these conditions will be analyzed individually to come up with a recommended therapeutic approach for each of them, applying pioneering pragmatism.

## 2. Pathophysiology and Classification of Non-Infectious Choroiditis: A Glimpse of the Essential

Until imaging investigation of the choroid became available thanks to indocyanine green angiography (ICGA), choroidal non-infectious inflammatory diseases were ill-understood and difficult to appraise. A first attempt at classification was undertaken in 1995 under the terminology of “white dot syndromes” (WDS) [17]. This classification, simply based on similar fundus appearance of lesions, was not only inappropriate but at the origin of much confusion during the 3 decades that followed by grouping diseases not belonging together [18].

**Figure 1:**
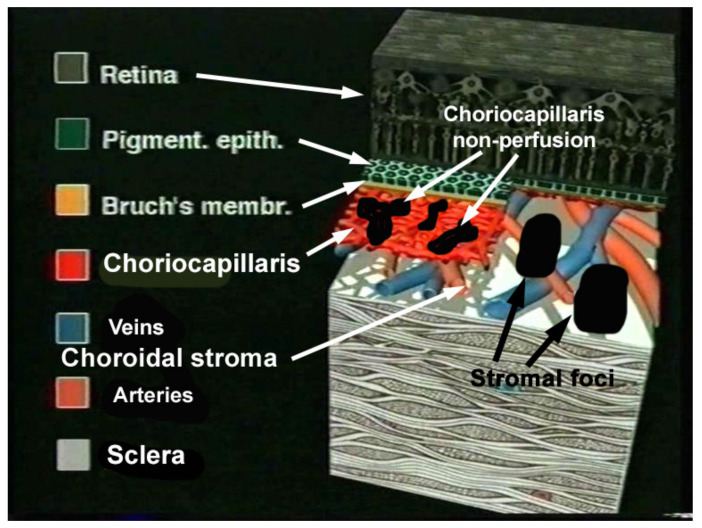
Cartoon illustrating disease mechanisms in non-infectious choroiditis. Choriocapillaris non-perfusion in case of choriocapillaritis and stromal foci in case of stromal choroiditis.

**Figure 2:**
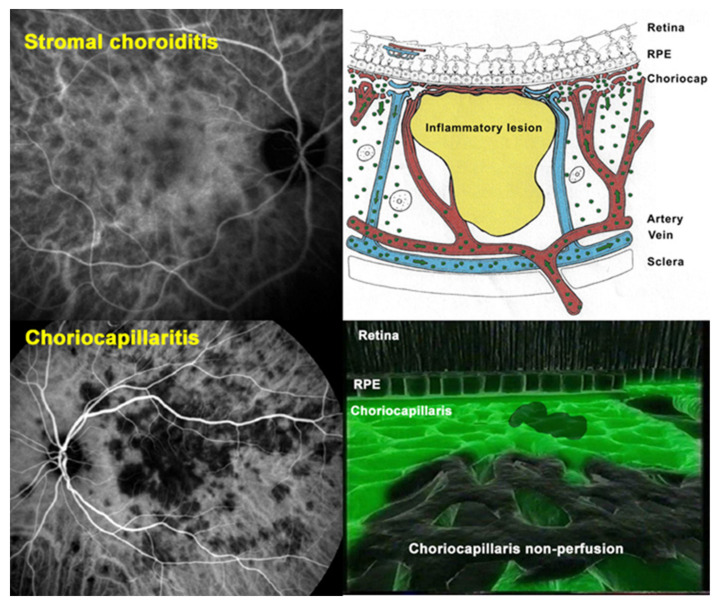
ICGA signs differentiating stromal choroiditis from choriocapillaritis. Top two pictures showing regular round hypofluorescent dark dots (HDDs) (**left**) and cartoon explaining the mechanism characterized by absence of diffusion of the ICGA dye impaired by the presence of stromal foci (**right**). Bottom two pictures showing geographical confluent hypofluorescent areas explained by absence of dye in choriocapillaris due to non-perfusion.

Once the choroid could be explored with substantial precision by ICGA [19], it became clear that there were at least two main mechanisms and patterns of non-infectious choroiditis, (a) stromal infiltration by inflammatory foci on one side (stromal choroiditis) and (b) choriocapillaritis producing inflammatory choriocapillaris non-perfusion on the other side [20] (Figure 1 and Figure 2).

Obviously, these diseases should not be classified together as they used to be in the past under the denomination of WDS. Choriocapillaritis entities include PICCPs [6], called primary because the trigger at the origin of ailment is unknown distinguishing them from so-called secondary forms where the trigger is identified such as ASPPC [14] or TB-SC [12,13]. The suspected triggers in PICCPs could be unidentified viruses, as a substantial number of patients present flu-like episodes before the ocular involvement [7]. PICCPs encompass conditions with diverse disease severity reaching from self-resolving diseases, such as MEWDS, to severe conditions leaving irreversible chorioretinal scars if not given prompt and aggressive treatment. The diverse spectrum of PICCPs is most probably conditioned by the size of the vessels affected by the vaso-occlusive process and by its severity. In stromal choroiditis the disease mechanism is fundamentally different and results from an autoimmune reaction to stromal antigens inducing progressive infiltration of the stroma that subsequently spills over to the adjacent structures. The two main diseases, VKH and HLA-A29-BRC, present a similar stromal choroidal involvement with the latter having a less aggressive course [21].

**Figure 3:**
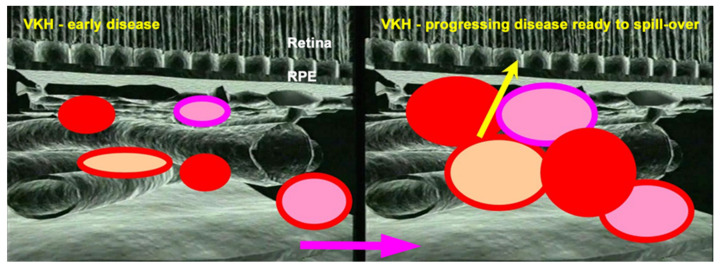
VKH stromal choroiditis. Cartoons show how VKH disease is progressing in the choroidal stroma (crimson arrow) before its spill-over to neighboring structures such as the retina (yellow arrow).

Another crucial difference between VKH disease and HLA-A29-BRC is the fact that the latter presents in parallel retinal inflammation unrelated to the choroiditis [21]. The precise classification of non-infectious choroiditis is not only a purely semantic preoccupation but, being aware of the diverse disease mechanisms will have an impact on the therapeutic intervention. For instance, in VKH the origin of inflammation develops exclusively from the stromal choroid with damage to other eye structures occurring only secondarily due to spill-over inflammation from the choroid (Figure 3). Therefore, it has to be borne in mind that systemic immunosuppressive therapy has a relatively easy access to this highly vascularized area and intraocular delivery of immunosuppressants is inadequate, having limited effect on the choroid. 

Similarly, choroidal disease in HLA-A29-BRC responds relatively well to immunosuppressive therapy, whereas retinal disease is more resistant because of the blood-ocular barriers [21].

## 3. Diagnostic and Monitoring Methods: A Summary of the Relevant Techniques

### 3.1. Invasive Methods

#### 3.1.1. Indocyanine Green Angiography (ICGA)

ICGA is the gold standard imaging modality for visualizing the choroid in a global fashion, as it allows us to detect infrared choroidal fluorescence, bypassing the RPE barrier [22]. Furthermore, the ICG molecule is strongly bound (98%) to large size blood proteins constituting a large molecular complex with a molecular weight of 60,000 to 80,000 Daltons (d) which remains intravascularly except at the level of the choriocapillaris from which it egresses through the large fenestrations to fill the choroidal stroma. ICGA has helped to better understand the pathophysiology of non-infectious choroiditis showing geographical hypofluorescent dark areas often confluent in choriocapillaritis, the expression of choriocapillaris non-perfusion of variable severity, different extension and patterns [23] (Figure 1 and Figure 2). On the other hand, in stromal choroiditis the angiographic translation of stromal foci consists of regular, round, evenly distributed hypofluorescent dark dots (HDDs) in case of primary stromal choroiditis entities where inflammation originates primarily within the choroid such as VKH and HLA-A29 BRC, whereas in secondary choroiditis, a chance location of a systemic disease such as sarcoidosis, the hypofluorescent areas are of different sizes with a random distribution. 

One advantage of ICGA for choriocapillaritis consists of the fact that it can show very limited end-capillary patchy hypofluorescence/non-perfusion as it is occurring in MEWDS. These are vessels with no or limited flow, therefore not accessible to optical coherence tomography (OCT) angiography (OCT-A) analysis. These patchy ICGA hypofluorescent lesions are better delineated in the late phases of angiography [8]. Detailed analysis and description of ICGA signs in choriocapillarites have been published previously [8,23].

**Figure 4:**
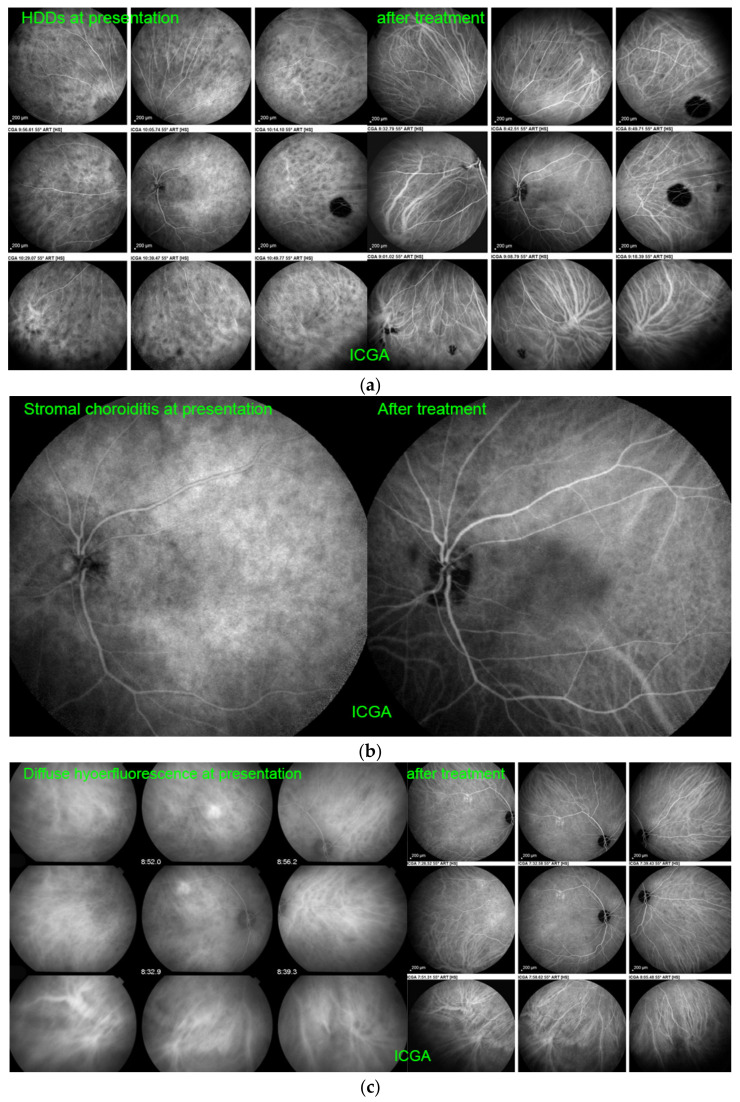

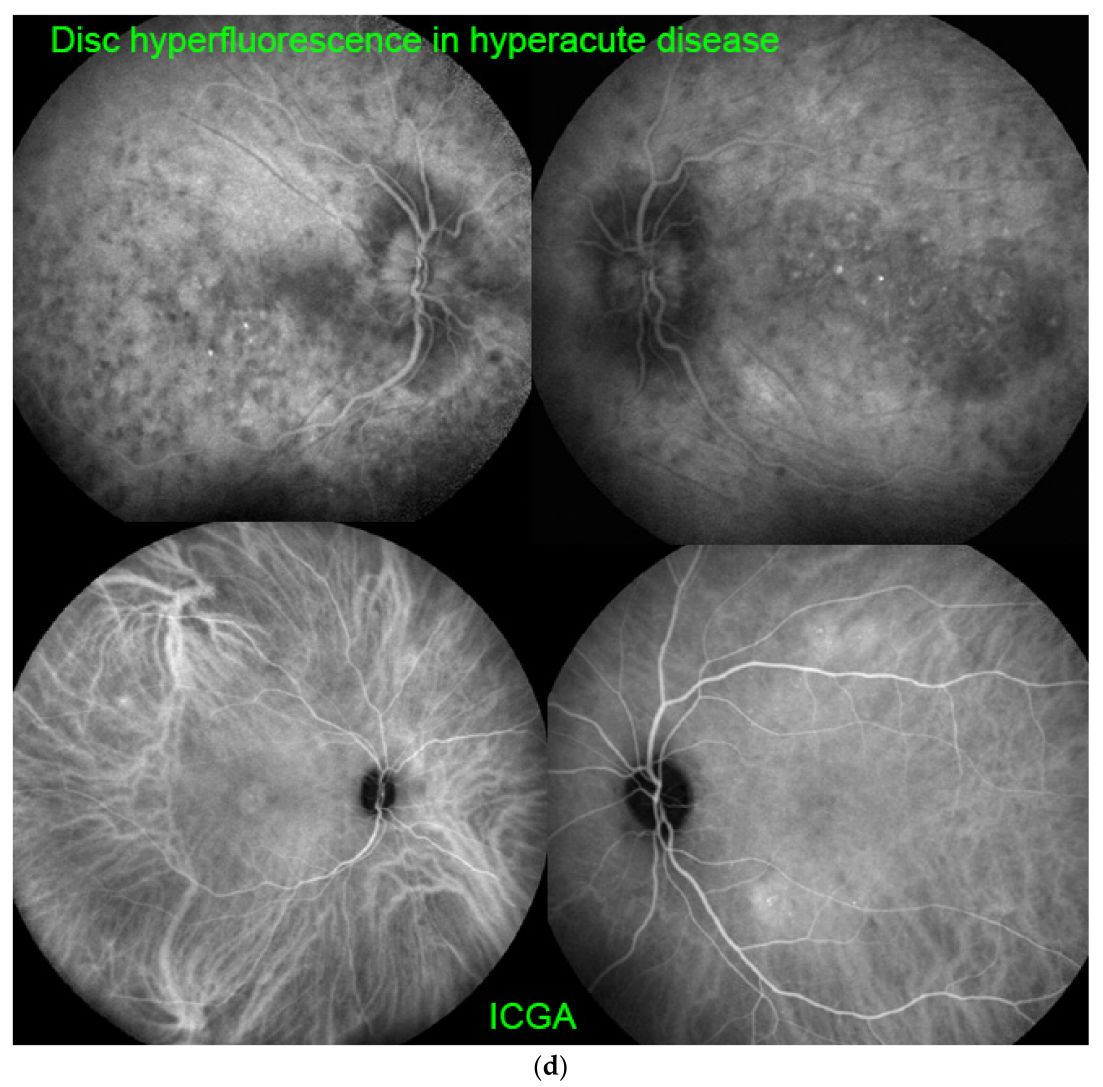
The 4 main ICGA signs in stromal choroiditis: (**a**) hypofluorescent dark dots (HDDs) in a case of HLA-A29-BRC stromal choroiditis. Numerous HDDs at presentation (**left 9 frames**) with choroidal vessels almost undistinguishable. After triple immunosuppressive treatment, most HDDs have disappeared and choroidal vessels are again well visible (**right 9 frames**). (**b**) fuzzy-indistinct choroidal vessels in a case of HLA-A29-BRC stromal choroiditis. No choroidal vessels can be distinguished hidden by HDDs and diffuse hyperfluorescence taken at presentation (**left frame**). After triple immunosuppressive therapy, choroidal vessels are again visible and most HDDs and diffuse hyperfluorescence have disappeared (**right frame**). (**c**) diffuse late hyperfluorescence in a case of VKH stromal choroiditis. Diffuse hyperfluorescence erases HDDs some of which are still distinguishable (**left 9 frames**) Note also the fuzzy leaking choroidal vessels (**left picture of 9 frames**). After triple immunosuppressive treatment diffuse hyperfluorescence has decreased as well as HDD (**right 9 frames**). Note that choroidal vessels are again well delineated (**right picture of 9 frames**). (**d**) ICGA disc hyperfluorescence in a case of acute VKH disease: bilateral ICGA hyperfluorescent discs at presentation (**top 2 frames**); note also HDDs and indistinct choroidal vessels. After triple immunosuppressive treatment, discs are again hypofluorescent, HDDs have resolved and choroidal vessels have recovered their normal aspect. As a rule, optic discs are always hypofluorescent on ICGA, except in severe choroidal inflammation.

ICGA is equally important for stromal choroiditis. In the diagnosis of initial-onset VKH, ICGA displayed 90–100% sensitivity for identification of disease-defining signs in multiple studies [24,25,26]. In these studies, the semiology of ICGA in initial-onset VKH disease has been precisely established. Four main ICGA signs have been defined, including hypofluorescent dark dots [HDDs], indistinct fuzzy choroidal vessels, diffuse late choroidal hyperfluorescence (partially hiding/erasing HDDs) and disc hyperfluorescence (Figure 4a–d). Enhanced-depth imaging OCT (EDI-OCT), measuring choroidal thickness, had a slightly lower sensitivity for identifying diffuse choroiditis, but its sensitivity was also above 90% in acute disease [27]. The major drawback of currently available imaging modalities such as EDI-OCT and OCT-A is that they do not show peripheral involvement; however, new generations of these instruments may overcome this disadvantage. In chronic VKH disease, EDI-OCT is less reliable than ICGA in detecting choroiditis, because the choroidal thickness is globally reduced. Therefore, EDI-OCT does not always detect disease reactivation. As far as therapeutic management is concerned, ICGA plays a crucial role in monitoring treatment efficacy and in the readjustment of therapy [28].

#### 3.1.2. Fluorescein Angiography (FA)

Fluorescein angiography, obviously, has a less important role in the appraisal of non-infectious choroiditis, as the inflammatory events take place in the choroidal compartment to which FA has limited access because of the retinal pigmentary epithelium (RPE) which is a screen to fluorescein fluorescence. In choriocapillaritis there is access to choroidal FA fluorescence only during the first 60 s of the angiogram when the intravascular quantity/concentration of fluorescein is very elevated showing choriocapillaris non-perfusion or perfusion delay. This sign allowed August Deutman to give the appropriate explanation for APMPPE, choriocapillaris non-perfusion rather than an RPE pathology, previously thought to be at the origin of the disease [29]. In some severe cases of PICCPs, FA shows late hyperfluorescence due to pooling in the late angiographic frames that can be interpreted as a reactive dilatation and exudation from inner retinal vessels secondary to a profound ischemia of the outer retina [30]. In stromal choroiditis, FA is useful in the early acute phase of VKH disease showing exudative retinal detachments with subretinal fluid and hyperfluorescent pinpoints through which fluid is leaking from the choroid. However, in HLA-A29 BRC, FA is useful throughout the disease course to follow the retinal component of inflammation and plays a monitoring role in disease evolution and response to therapy [21].

### 3.2. Non-Invasive Methods

#### 3.2.1. Fundus Autofluorescence (FAF)

**Figure 5:**
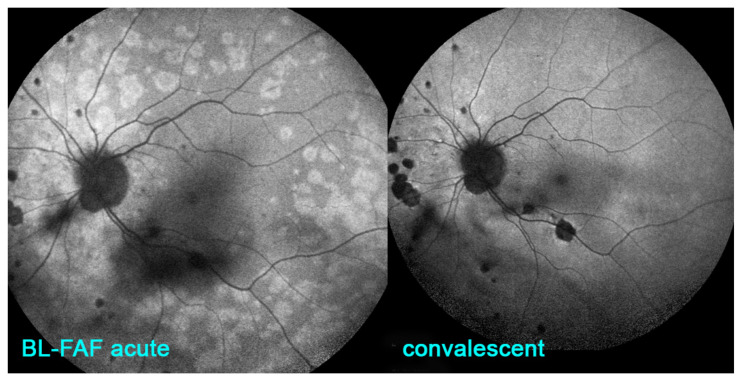
Blue-light fundus autofluorescence (BL-FAF) for the follow-up of MEWDS and Multifocal choroiditis (MFC). BL-FAF is a follow-up modality showing increased autofluorescence from lipofuscin contained in RPE cells in diseased areas where the photoreceptor outer segment “screen” is lost. Acute phase of recurrence of MFC (**left frame**) showing clear hyperautofluorescent areas that fade out progressively after triple immunosuppressive therapy (**right frame**).

Blue-light fundus autofluorescence (BL-FAF) is a non-invasive imaging method, which can demonstrate RPE and photoreceptor pathology even at an early stage of disease. The autofluorescence signal derives from the normal lipofuscin accumulation in the RPE cytoplasm. In PICCPs, patchy and/or geographic hyperautofluorescence is the main finding caused by the loss of the photoreceptor outer segments [31]. This reduces the photoreceptor screen allowing to better see the BL-FAF signal originating from the lipofuscin contained in RPE cells (Figure 5).

In non-infectious choroiditis this modality is principally applied to conditions causing damage to the photoreceptor outer segments, i.e., choriocapillaritis but is inappropriate for stromal choroiditis. The non-invasive character of this exam makes it very useful in diagnosis but more importantly in the follow up of the inflammatory choriocapillaropathies and constitutes a measurement parameter of therapeutical intervention. In some cases, such as MFC and MEWDS, BL-FAF can indeed serve as a substitute to ICGA, in the follow-up of patients [32].

#### 3.2.2. Spectral Domain-Optical Coherence Tomography (SD-OCT) and Enhanced Depth Imaging OCT (EDI-OCT)

**Figure 6:**
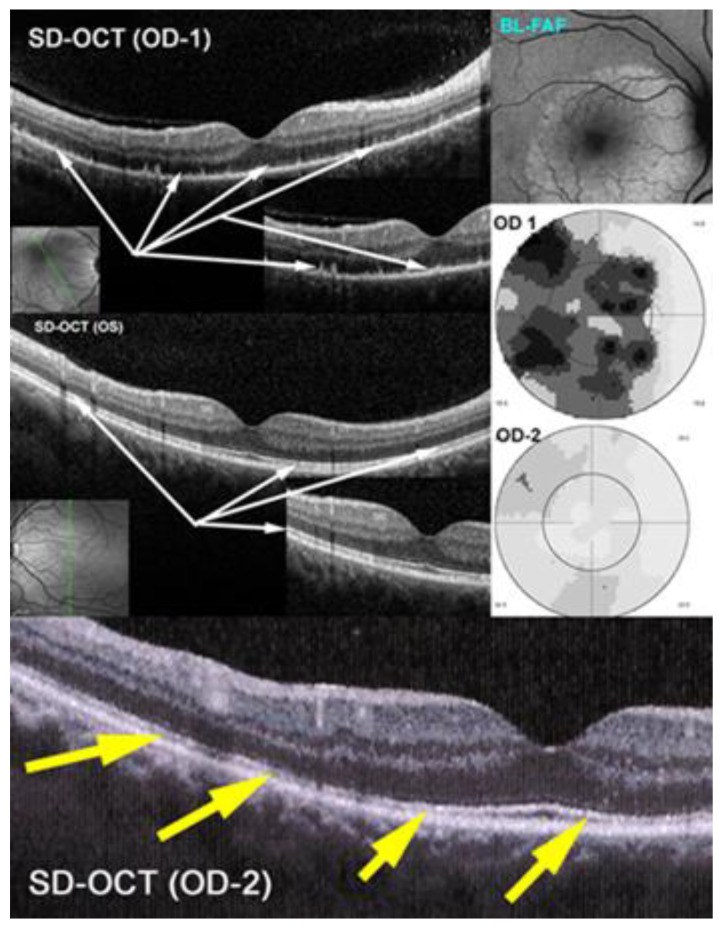
Spectral domain optical coherence tomography (SD-OCT) in a case of ASPPC choriocapillaritis. SD-OCT gives morphological information on photoreceptor outer segment damage. Top left SD-OCT (OD) figure shows extensive loss of photoreceptor outer segments with only a few spicules left (white arrows) corresponding to hyperautofluorescence on BL-FAF (**top right**) and corresponding to extended visual field loss (OD-1). The middle SD-OCT (OS) image shows a normal line of photoreceptors in the left non-affected eye. The lower image (SD-OCT (OD-2) shows reconstitution of the photoreceptor outer segments (yellow arrows) 7 weeks after initiation of treatment with normalization of the visual field (OD-2).

Spectral-domain optical coherence tomography (SD-OCT) is a non-invasive and easily repeatable method showing the damage, loss and regeneration of photoreceptor outer segments [33] (Figure 6). As choriocapillaris non-perfusion and its consecutive ischemia directly affects the outer retina, especially the photoreceptors, OCT demonstrates the morphological effect of choriocapillaritis on the outer retina in PICCPs by showing the extent of secondary damage to the photoreceptor outer segments (Figure 6).

The findings can be minimal, such as interruption or swelling of the IS/OS ellipsoid zone in some cases of MEWDS to extended zones of atrophy in serpiginous choroiditis. OCT is also helpful in detection of inflammatory choroidal neovascularization (ICNV) or macular oedema. In HLA-A29-BRC stromal choroiditis SD-OCT was useful to show the posterior pole retinal involvement [34].

**Figure 7:**
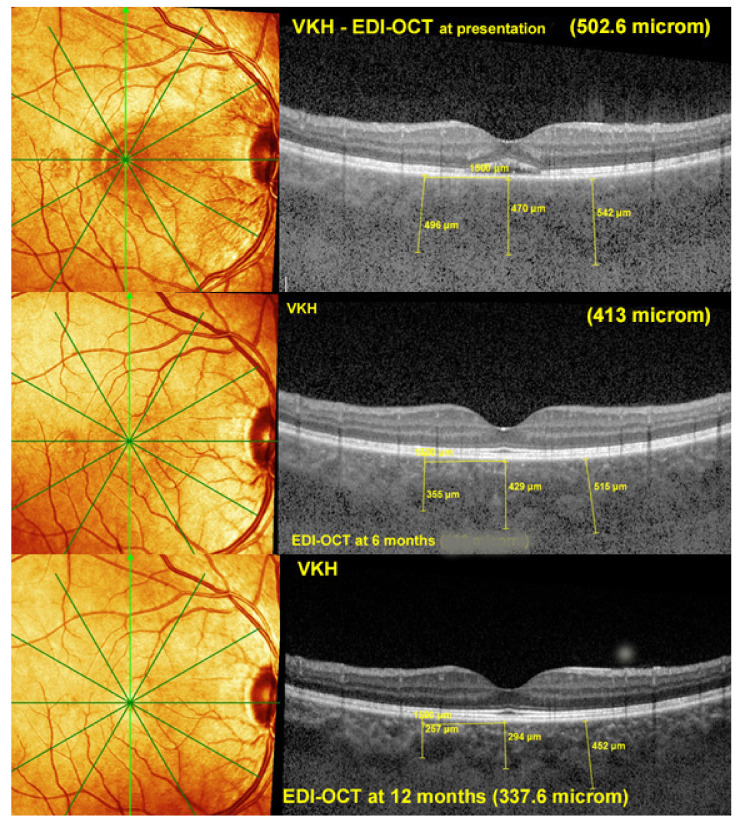
Enhanced depth imaging OCT (EDI-OCT) measures choroidal thickness (yellow calipers) and allows us to monitor stromal choroiditis. Thickness at presentation (**top image**) amounts to 502.6 µm, to decrease to 433 µm at 6 months (**middle image**) and to 337.6 µm (**bottom image**).

Enhanced depth imaging OCT (EDI-OCT) allows the quantitative measurement of choroidal thickness in inflammatory choroidal stromal choroiditis and represents a monitoring modality to follow the evolution of stromal inflammation/infiltration [35,36] (Figure 7). A major limitation is the individual variation in choroidal thickness due to physiological variables such as age, ethnicity, gender, axial length, refractive error, intraocular pressure, and systolic blood pressure [37].

At presentation in initial-onset stromal choroiditis such as HLA-A29-BRC and VKH disease the choroid is substantially thickened and gradually decreases under treatment. In case of chronic and insufficiently treated cases the choroid can become thinner than normal. EDI-OCT gives the same information as ICGA but is limited to the posterior pole. It is less accurate than ICGA but can be used as a substitute when ICGA is not available [27]. In post-acute and chronic disease there is a mixture of thinned and still thickened choroid rendering the measurement less reliable.

#### 3.2.3. OCT Angiography (OCT-A)

**Figure 8:**
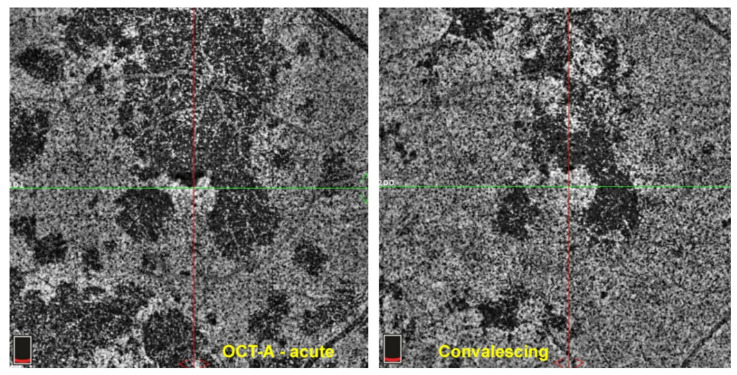
Optical coherence angiography (OCT-A) in a case of APMPPE/AMIC. Choriocapillaris non perfusion (drop out) at presentation (**left image**) with progressive regression of choriocapillary drop-out in the convalescent phase (**right image**).

Optical coherence tomography angiography (OCT-A) is a non-invasive imaging modality that has recently been added to our array of investigations of the retina and inner choroid [38]. It detects flow in vessels of the retina and choroid. In case of choriocapillaritis entities it shows capillary drop out except in end-choriocapillary vessels which cannot be imaged by this method as there is absence of significant flow. These vessels, thought to be at the origin of MEWDS, are not detected by OCT-A, unless MEWDS is severe, and hence drop-out cannot be demonstrated. Therefore, this apparent lack of drop-out has been misinterpreted as choriocapillaris integrity, when it is only due to the fact that the methodology is inappropriate and cannot be applied to this situation. In case of choriocapillaris drop-out of larger areas caused by involvement of larger vessels such as in APMPPE, MFC and SC, it is a useful additional imaging tool for the evaluation of the extent of lesions [39] (Figure 8). For most instruments presently used in routine practice, the imaging is limited to the central area of the posterior pole with new instruments in the pipeline progressively giving access to more peripheral areas. The patchy absence of choriocapillaris flow signal (choriocapillaris drop-out) shown by OCT-A in PICCPs does not contribute to a substantially better information than ICGA, except that it is non-invasive.

#### 3.2.4. Visual Field Testing and Microperimetry

Visual field testing shows functional impairment and corresponds to BL-FAF hyperautofluorescence and ICGA hypofluorescence and is useful for the functional follow-up. In subtle choriocapillaris non-perfusion as seen in MEWDS of limited involvement visual field testing may be normal and microperimetry is more sensitive to identify decrease in retinal sensitivity.

## 3.3. Imaging Biomarkers of Inflammation in Non-Infectious Choroiditis and Monitoring of Therapeutical Intervention

In order to evaluate the degree of inflammatory activity and to determine its response to therapy, objective parameters or biomarkers of the level of inflammation are in crucial need. For choriocapillaritis entities, the most reliable, useful and objective assessment modality is ICGA at presentation and for monitoring of the evolution after introduction of treatment, although it is only giving qualitative information. The drawback of ICGA is that it is an invasive procedure that cannot be repeated too often. Therefore, after being performed in parallel with ICGA, for follow-up purposes, in most choriocapillaris cases, evolution can also be monitored by BL-FAF, which is also a substitute when ICGA is unavailable [32]. SD-OCT confirms morphologically the improvement or not of lesions after therapeutical intervention. At present, OCT-A, with the routinely used instruments nowadays, is still less accurate than ICGA to mark non-perfusion and is limited to a small posterior pole area [39].

**Figure 9:**
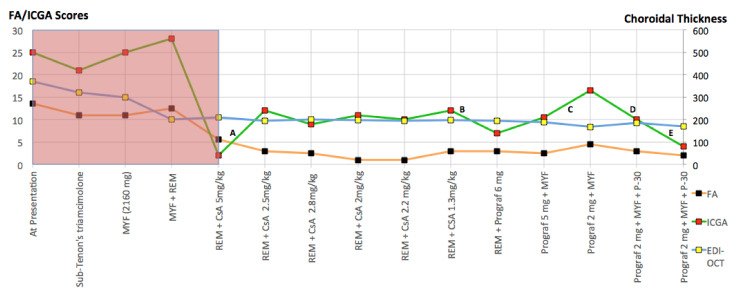
Fine tuning of therapy in a BRC patient where treatment had to be adjusted because of side-effects of ongoing therapy. (A) ICGA increased score showed no control of the disease which responded better after CsA changed to Tacrolimus (Prograf^®^) (B) as shown in decreased ICGA score. Reduced Prograf^®^ dosage provoked again increase of choroiditis (C) but the combination of low dose Prograf^®^, Myfortic^®^ and 30 mg of prednisone was the combination that controlled the disease again. with decrease of ICGA score (D and E). MYF = Myfortic^®^, mycophenolic acid. REM = Remicade^®^, infliximab, anti-TNF-α agent. CsA = cyclosporine. Prograf^®^ = tacrolimus. P = prednisone. Pink shadowed area accounts for disease evolution and treatment combinations before disease was under control. (Reprinted from J Curr Ophthalmol 2019; 31:180–187).

The role of ICGA is much more important for stromal choroiditis, as it appears as a very sensitive and semi-quantitative biomarker of stromal inflammation and is very reactive to show the impact or absence of impact of treatment modifications [28] Figure 9. shows fine-tuning of therapy in a BRC patient based on semi-quantitative scoring of FA/ICGA findings [40] and EDI-OCT choroidal thickness measurements.

For VKH it is the only modality that allows a precise follow-up, fine-tuning of immunosuppressive treatment [41] and is able to show complete absence of subclinical choroidal inflammation, which is needed when considering withdrawal of treatment [42]. In case of unavailability of ICGA, EDI-OCT can be used as a substitute with less precision [27].

In HLA-A29-BRC, ICGA precisely monitors choroiditis and can be substituted by the less accurate EDI-OCT. It has however to be completed by FA and retinal SD-OCT to follow retinal disease in particular retinal vasculitis [34]. Visual field testing has also a role in monitoring HLA-29-BRC.4. 

## 4. General Principles of Immunomodulatory Therapy for Non-Infectious Choroiditis: A Brief Overview of the Main Agents Used

Treatment strategy for non-infectious choroiditis depends on the form of disease and its severity. This is true for PICCPs. For example, MEWDS, can usually be followed and observed without treatment. Recommendation for APMPPE in textbooks is also forbearance of treatment. However, most of our APMPPE cases were pronounced and needed at least systemic corticosteroid therapy. In case of MFC and SC, dual or even triple immunosuppression is recommended. The diverse evolution of each single condition demands an individualized approach guided by close imaging follow-up and re-orientation of therapy if needed. Nowadays, we have an arsenal of imaging methods to help us determine whether there is a stability or deterioration of a disease. For example, if ICGA and/or BL-FAF follow-up of MEWDS shows a deterioration, therapeutical intervention can sometimes become necessary. As far as stromal choroiditis is concerned including VKH and HLA-A29 birdshot choroiditis (HLA-A29-BRC) aggressive dual and even triple immunosuppression is usually needed. Hereunder we present a non-exhaustive summary of the agents usually used in the literature and the strategy used in our institution.

### 4.1. Corticosteroids

Corticosteroid therapy remains the first line therapeutical option for non-infectious choroiditis, as it is the single most rapidly acting inflammation suppressive agent. However, as for the whole of uveitis management, we and others tend to limit the duration and amount of its use because of the morbidity of long-term corticosteroid administration. In some rare instances, local corticosteroid treatment may be used, especially when involvement is predominant in one eye and can be sufficient to reverse the pathology or delay systemic therapy in some cases [43]. For severe cases of PICCPs and stromal choroiditis, oral treatment generally starts at 1 mg/kg/day with a relatively rapid tapering over 4–6 months. In hyperacute diseases, such as VKH, treatment can be initiated with a 3-day course of 1 g of intravenous methylprednisolone. For secondary choriocapillaritis such as TB-SC and ASPPC, specific antibiotic therapy should be given in addition to corticosteroid therapy. Side effects of corticosteroids include, among others, promotion of infectious conditions that should be eliminated, hyperglycaemia, excitement/sleeplessness, restlessness, osteoporosis, osteonecrosis, weight gain and the rare but deleterious side effect of psychotic evolution. Dietary precautions should be taken to limit salt and carbohydrate intake. We usually conduct immunosuppressive therapy in concordance with the internist or immunologist. Ocular side effects consist mainly of steroid-induced ocular hypertension and/or glaucoma, posterior subcapsular cataract development and central serous chorioretinopathy [44,45].

### 4.2. Immunomodulatory/Immunosuppressive Agents

As corticosteroid therapy can provoke many deleterious and dangerous side effects, especially when used in the long run and as the main therapy, the switch to immunomodulatory/immunosuppressive (IS), less toxic agents must be planned and rapidly resorted to in case of severe inflammation and/or lack of response to corticosteroids. In many cases we do not really switch but use first-line dual steroidal and non-steroidal immunosuppression (one or two IS agents) in order to be able to taper corticosteroids as rapidly as possible. The use of such “corticosteroid-sparing” agents is much more current presently in ocular inflammatory diseases than the use of corticosteroid monotherapy that was practiced in the past. As for corticosteroids, it is important to rule out infectious causes of uveitis before the use of IS agents. Below is a brief overview of the usual agents used in non-infectious or immune-mediated choroiditis (Table 1).

#### 4.2.1. Antimetabolites

Azathioprine (Imuran^®^, Imurek^®^) is a purine analogue which interferes with DNA and RNA synthesis and is a cytostatic drug for T-cells. Azathioprine is a “slow starter” which means that it needs 8 weeks to 4 months to be fully active. Azathioprine is transformed into inactive metabolites by the enzyme thiopurine S-methyltransferase (TPMT). The enzyme is absent in approximately 1/300–400 patients, leading to severe toxicity. Therefore, at the start of treatment there should be great caution to detect unusual intolerance to the drug. Some institutions test for the absence of TPMT before administering the drug [46]. In our practice we usually start with a high dosage of 2.25–2.75 mg/kg/day but always less than 3 mg/kg, in order to detect relatively quickly whether the drug is effective or not, comparing to the strategy of progressive increase in the dosage. Blood counts and hepatic laboratory tests should be performed every 2 months at the initiation of therapy and about every 4 months thereafter as adverse effects include bone marrow suppression and hepatic toxicity [47]. Milder side effects are upper gastrointestinal symptoms. Azathioprine can be at the origin of birth defects but is considered moderately safe in pregnancy and lactation. Azathioprine, together with cyclosporine, tacrolimus and TNF-α inhibitors are part of the World Health Organization’s list of essential medications [48].

Mycophenolate mofetil (MMF, Cellcept^®^) has a strong cytostatic effect on T and B lymphocytes by inhibiting the enzyme inosine-5′monophosphate dehydrogenase, and stopping the purine biosynthetic pathways [49] and so decrease antibody production of B-cells. It is also characterized as a “slow-starter” with full activity only several weeks after onset of therapy. The recommended dosage is 1–3 g per day, and it is better tolerated than azathioprine, even with maximal doses. MMF has minimal side effects such as gastrointestinal discomfort, bone marrow depression and disturbed liver function tests [50].

Mycophenolic acid/sodium (Myfortic^®^) is an alternative form of mycophenolate and is better absorbed than MMF. It has a better gastro-intestinal tolerance as it comes as enteric coated tablets [51]. It is given at the dosage 720 mg twice a day. Both forms should be stopped during pregnancy and lactation. As for other conventional immunosuppressants, duration of treatment should usually be continued for at least two years in non-infectious choroiditis/uveitis.

Methotrexate (MTX)

Methotrexate is a folate analogue that inhibits several enzymes, including dihydrofolate reductase, involved in purine synthesis and hence producing a reduction in DNA and RNA synthesis which suppresses proliferation of rapidly dividing cells such as leucocytes [52].

Although it has been administered in uveitis since its FDA approval in 1953, it is still an off-label use as many other immunosuppressants listed here. It has a relatively narrow therapeutic index with the main side effects being liver toxicity, gastrointestinal upset, leukopenia due to bone marrow suppression and hair loss. Its intraocular penetration is not known. It is fully active after 5–7 weeks. The recommended dosage is 7.5 to 25 mg given once weekly either orally or subcutaneously, the latter route possibly causing less gastro-intestinal side-effects. MTX use in non-infectious choroiditis has been reported, among others, in the treatment of MFC and SC [53,54] as well as HLA-A29 BRC [55]. Intraocular administration has also been described [56]. We rarely use MTX due to its narrow therapeutic index and the unknown intraocular penetration.

#### 4.2.2. Calcineurin Inhibitors (CI)

CI block T lymphocytes by suppressing the production of IL-2, a major enhancer for T-cell activation and recruitment. Cyclosporine (CsA) and tacrolimus (FK 506) are the two most commonly used agents of this category. Unlike antimetabolites, this class of immunosuppressive agents achieves clinical efficacy within 1–2 weeks and allows us to begin tapering corticosteroids relatively rapidly, bridging the time lag of 2–4 months until antimetabolites reach their full efficacy [57] 

Cyclosporine (CsA) (Sandimmun^®^)

In ophthalmology, CsA was first shown to be efficient in experimental autoimmune uveitis [58] and subsequently in uveitis in humans [59]. 

The starting dose of CsA is 3–5 mg/kg/day. We tend to start with the higher dosage of 4.5–4.8 mg/kg rather than to increase dosage progressively, in order to know as soon as possible whether a therapeutical impact is obtained. Taper is usually started after 6–8 weeks, and discontinuation of therapy after 6–9 months is usually the objective after the concomitantly administered anti-metabolite is fully active. When efficacy is suboptimal, whole blood drug levels 6 hours post-dose rather than pre-dose trough levels should be measured before concluding that CsA is ineffective [60,61]. Extreme caution must be applied to monitor renal function and arterial hypertension, the major adverse effects. Other side effects are dyslipidemia, hirsutism and gingival hyperplasia [62]. When performing a literature search using the terms of cyclosporine and, respectively, birdshot, VKH disease, serpiginous choroiditis, idiopathic multifocal choroiditis punctate inner choroidopathy, more than 25 articles reported the use of CsA.

Tacrolimus (FK 506) (Prograf^®^)

Tacrolimus has the same mechanism of action as CsA, targeting T lymphocytes by suppressing the stimulating growth factor interleukin-2 (IL-2) but appears to present a better toxicity profile with at least similar if not better efficacy [63,64]. The initial dosage for Tacrolimus is 0.05–0.15 mg/kg per day. Similar to CsA and other immunosuppressants such as MMF, in case of lack of efficacy whole blood drug monitoring should be performed by measuring trough levels 12 hours post-dose with optimal trough levels of 5–10 µg/L. Side effects are similar to cyclosporine, as far as nephrotoxicity and neurological complaints are concerned but has a better profile concerning cardiovascular side effects, dyslipidemia, gingival hyperplasia and hirsutism. A serious side effect is the diabetogenic effect of tacrolimus. Very few reports on treatment of non-infectious choroiditis were found in the literature and limited to stromal choroiditis (HLA-A29-BRC and VKH disease) [65,66]. This indicates that tacrolimus is still not currently considered for non-infectious choroiditis.

Long-term, retrospective, multicenter data on over 8500 patients with non-infectious ocular inflammatory diseases, including uveitis, published by the Systemic Immunosuppression Therapy for Eye Disease (SITE) Study Group, have shown control of inflammation at 12 months with corticosteroid-sparing success in 36% of patients treated with CsA, 47% with azathioprine, 55% with MMF, and 58% with MTX [67]. Thus, a significant proportion of patients with ocular inflammatory diseases may not adequately respond to these agents when used as a single corticosteroid-sparing IS agent, and switching between agents and/or combined use of agents may be required. As already mentioned, duration of treatment should usually be continued for at least two years in non-infectious choroiditis/uveitis. Even though alkylating agents, cyclophosphamide and chlorambucil, have been used for the treatment of severe refractory cases of non-infectious uveitis/choroiditis including SC [68], they are not currently preferred, if not completely abandoned, due to their potential serious adverse effects.

### 4.3. Biological Agents

This is a relatively new group of therapeutic agents that halt the inflammatory reaction by inhibiting different pathways and/or cells of the inflammatory cascade, including anti-TNFα agents (Infliximab, Adalimumab), anti-CD20 agent (Rituximab), IL-1 receptor antagonist (Anakinra) and IL-6 receptor antagonist (Tocilizumab) [69]. Dose, mechanism of action and side effects are detailed in Table 2. As with IS, active infections should be excluded in order to start treatment. In particular, it is crucial to rule-out a previous contact with Mycobacterium Tuberculosis by performing an interferon gamma release assay (IGRA), as the use of biological agents, especially anti-TNF-α agents, may be lethal in case of tuberculosis. There is limited and only anecdotal evidence of efficacy of biological immunomodulatory agents in choriocapillaritis and mostly TNF-α blockers have been used. Infliximab was shown to be efficient in severe uveitis [70] as well as in case reports and small series of patients in MFC and SC [71,72]. Biological agents have proven their efficacy in HLA-29 birdshot choroiditis and VKH [73,74]. The efficacy and safety of adalimumab has been proven in randomized controlled trials of non-infectious intermediate, posterior, and panuveitis in adults and is currently the only biologic agent approved for the treatment for the treatment of posterior/panuveitis in adults [75]. This is not an exhaustive review of all biological agents, not including agents that have not been used for non-infectious choroiditis, such as Golimumab, Cerolizumab-pegol and Canakinumab. More details on these substances can be found in [69].

Secondary choriocapillaritis entities such as TB-SC and ASPPC are rarely treated by biologic agents as the risk of activation of a latent infectious pathology is elevated, and biologic therapy should only be considered after effective control of infection with specific antibiotic therapy.

## 5. Treatments and Novel Therapeutic Approaches of Non-Infectious Choroiditis

### 5.1. Choriocapillaritis

#### 5.1.1. MEWDS

In textbooks, no particular treatment is recommended for MEWDS as it is usually a self-limited disease [81]. However, after the initial angiography coupled with BL-FAF, there should be a strict follow-up performed, using BL-FAF to document safe recovery. If this is not the case and worsening is noted, action should be taken, as it can be an atypically evolving case [82] or more probably the first episode of MFC as both diseases have similar presentations and sometimes the diagnosis of MFC can only be ascertained when the disease recurs [83]. In both cases systemic corticosteroid therapy should be initiated. If further worsening occurs a rapidly acting immunosuppressive agent such a calcineurin inhibitor should be added, followed by an anti-TNF-α biological agent in case of insufficient response. Fortunately, BL-FAF monitoring allows us to avoid repeat ICGA. 

#### 5.1.2. Acute Posterior Multifocal Placoid Pigment Epitheliopathy/Acute Ischemic Multifocal Choriocapillaritis (APMPPE/AMIC)

APMPPE was first attributed to the RPE as the primary site of tissue insult by Gass in 1968 [84]. Now we know that the origin of the disease is due to choriocapillaris non perfusion as for other PICCPs, thanks to work published by Deutman who correctly identified the primary site of involvement and named the disease acute multifocal ischemic choriocapillaritis [29] (Figure 8). Compared with other PICCPs, APMPPE/AMIC seems to be in the middle range of the spectrum of severity, but involvement can greatly vary from one case to the other. APMPPE/AMIC was at first considered as a self-limited disease as some cases can resolve without treatment, but it seems that more than 20% of cases have severe non-perfusion of choriocapillaris and treatment is necessary [9].

Although prognosis is generally good close follow up is recommended. Macula involvement should be an indicator to start a treatment as the progress of the disease can lead to significant vision loss. Fiore et al. have found in their retrospective study and review of the literature in 2009 that about 54% of APMPPE cases were treated while 71% of patients still were symptomatic in last follow-up [85]. Oral corticosteroid is the treatment of choice, starting with 0.8–1 mg/kg/day gradually tapered over several weeks, depending on imaging findings. In severe cases a second immunosuppressor may be needed, with tacrolimus/cyclosporine [86] or mycophenolate mofetil [9] having this role. Methotrexate is an alternative as described in case reports [87]. Anti-TNFa treatment has also been proposed by some specialists as a treatment for severe cases. El-Markaby et al. have described in their study 8 cases of severe APMPPE treated with Infliximab with significant improvement of vision at last follow-up [88]. Even though systemic treatment is mostly preferred for APMMPE, local treatment, such as subTenon’s injection of triamcinolone, intravitreal injection of triamcinolone and dexamethasone intravitreal implant, have also been described [89,90]. 

#### 5.1.3. Idiopathic Multifocal Choroiditis (MFC)

**Figure 10:**
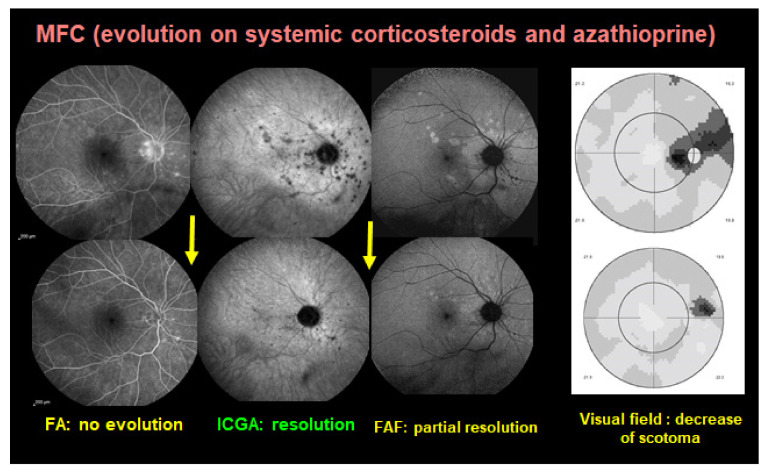
Monitoring of therapeutical intervention in idiopathic multifocal choroiditis. Episode of MFC recurrence analysed by multimodal imaging. Fluorescein angiography (**2 far left frames**) is marginally efficient to monitor therapy. ICGA (**middle left frames**) exactly identifies hypofluorescent lesions that regress after triple immunosuppressive therapy (**bottom image**). BL-FAF (**middle right frames**) is another modality to monitor treatment showing fading of hyperautofluorescence (**bottom image**) The far right column shows improvement of visual field after treatment (**bottom image**).

Idiopathic Multifocal Choroiditis (MFC) is a PICCP, predominant in healthy myopic women. The choriocapillaris non-perfusion in MFC resembles MEWDS although MFC has a recurrent course and is frequently complicated by ICNV (Figure 10). Punctate inner choroidopathy should be considered as part of the same pathology as MFC. Treatment of MFC is still under discussion and systemic treatment is not universally accepted. While some specialists consider systemic treatment only if ICNV is detected or if lesions are close to the fovea [91], De Groot et al. have shown in a recent study that immunomodulatory therapy significantly decreased the number of recurrences of MFC. They also concluded that systemic treatment decreased the number of anti-vascular endothelial growth factor (anti-VEGF) injections needed in the cases with ICNV [92], as it has been reported for other uveitic ICNV [93]. The number of immunosuppressors needed is also a debate amongst specialists. Goldberg et al. have demonstrated a single agent success of therapy for 81% of patients at 6 months, 76% at 1 year, and 95% at 2 years. However, they mentioned that 77% of patients required an increased dose of MMF and 21% of patients required a second immunosuppressive agent within 2 years to maintain quiescence [94]. In our experience, triple immunosuppressive treatment has halted the progression of disease, without recurrences in a three-year follow-up period [10]. Corticosteroid treatment is still the first choice, starting with 1 mg/kg/day, adding a rapidly acting immunosuppressant of the calcineurin inhibitor family (tacrolimus or CsA) in order to taper corticosteroids as soon as possible, also used to bridge the time span of 8–12 weeks until anti-metabolite immunosuppressants such as MMF and azathioprine are fully active. Biological agents can be used in cases insufficiently well controlled by classical immunosuppression or because of treatment intolerance. In case of associated inflammatory choroidal neovascularization (ICNV) more frequent in MFC than other choriocapillaritis entities, intravitreal anti-VEGF injections have to be added. A study by Feng L et al. has shown that fewer number of injections are needed comparing to patients with neovascular age related macular degeneration [95].

#### 5.1.4. Serpiginous Choroiditis (SC)

**Figure 11:**
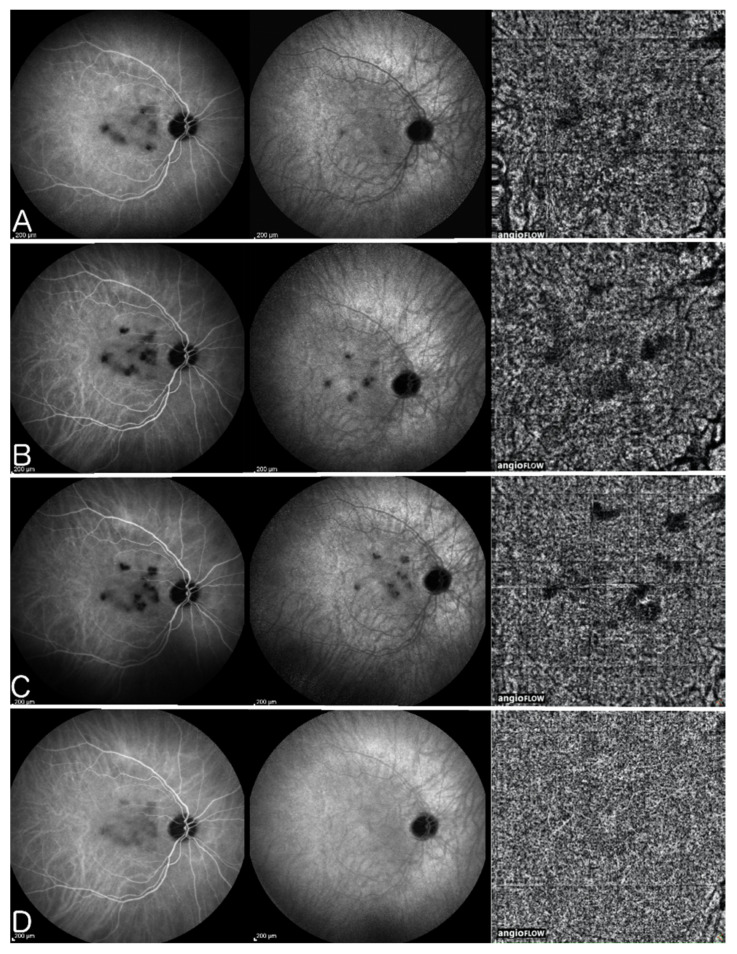
Serpiginous choroiditis—Parallel images of intermediate phase ICGA (**left column**), late phase ICGA (**middle column**) and 6 × 6 mm OCT-Angiography (**right column**) to monitor disease evolution and therapeutic intervention. ICGA of the right eye reveals macular hypofluorescent lesions better seen in the intermediate phase ICGA (**left column**) corresponding to choroidal hypoperfused areas. Lesions seen at presentation (**A**) increase in size and number in the right eye after 5 months (**B**) and 8 months (**C**) despite a posterior sub-Tenon’s triamcinolone injection performed at 5 months. The late phase ICGA frames (**middle column**) confirm the worsening of perfusion as hypofluorescence appears at 5 months (**B**) and persists at 8 months (**C**) despite the subTenon’s injection at 5 months. Three months after adding systemic cyclosporine to the treatment (**D**) there is a substantial decrease in hypofluorescence in the intermediate phase (**bottom left picture**) and disappearance of dark areas in the late ICGA frame (**bottom middle**). OCT-A pictures evolve in parallel with ICGA frames with faint dark areas visible at presentation (**A, right**), more clearly visible at 5 months (**B, right**) and 8 months 2016 (**C, right**) and disappearing 3 months after additional cyclosporine treatment. Both ICGA and OCT-A monitor the evolution with a better precision on ICGA [39].

Serpiginous choroiditis is the most severe of the PICCPs. As it is involving larger choriocapillaris vessels it can progress relentlessly in a creeping fashion if not treated rapidly and vigorously (Figure 11). Before initializing treatment, an IGRA test should be performed to rule out tuberculosis related SC. If immunosuppressive treatment is not administered as soon as possible, the condition will progress, causing irreversible chorioretinal atrophy and severe functional impairment. Aggressive triple immunosuppressive treatment (prednisone 1 mg/kg/day, cyclosporine 5 mg/kg/day and azathioprine 1.5–2 mg/kg/day) was already proposed by Hooper PL and Kaplan HJ in 1991 [96] who mentioned rapid resolution of disease in all patients included in their study, which was confirmed by later studies [97,98]. Intravenous pulse methylprednisolone has been used in episodes of macula-threatening SC occurring during maintenance therapy with azathioprine and CsA [99]. Venkatesh et al. have prospectively randomized 30 patients with macular or macula-threatening SC to treatment with 3-day pulse corticosteroid or pulse cyclophosphamide or a combination of both and observed a complete healing of the lesions at a median duration of two weeks in each group. However, relapses were seen after tapering oral corticosteroids, also in patients who had received 3-day cyclophosphamide pulses initially [100]. The severity of the disease is well recognized and explains that in some centers even more toxic agents such as chlorambucil have been used [101].

With the introduction of anti-TNF-α agents in our arsenal, studies have shown the effectiveness of infliximab and adalimumab in halting the progression of the SC [70,72,102]. Noda et al., however, reported limited efficacy of adalimumab in a patient with SC serpiginous choroiditis [103].

#### 5.1.5. Tuberculosis Related Serpiginous Choroiditis (TB-SC)

**Figure 12:**
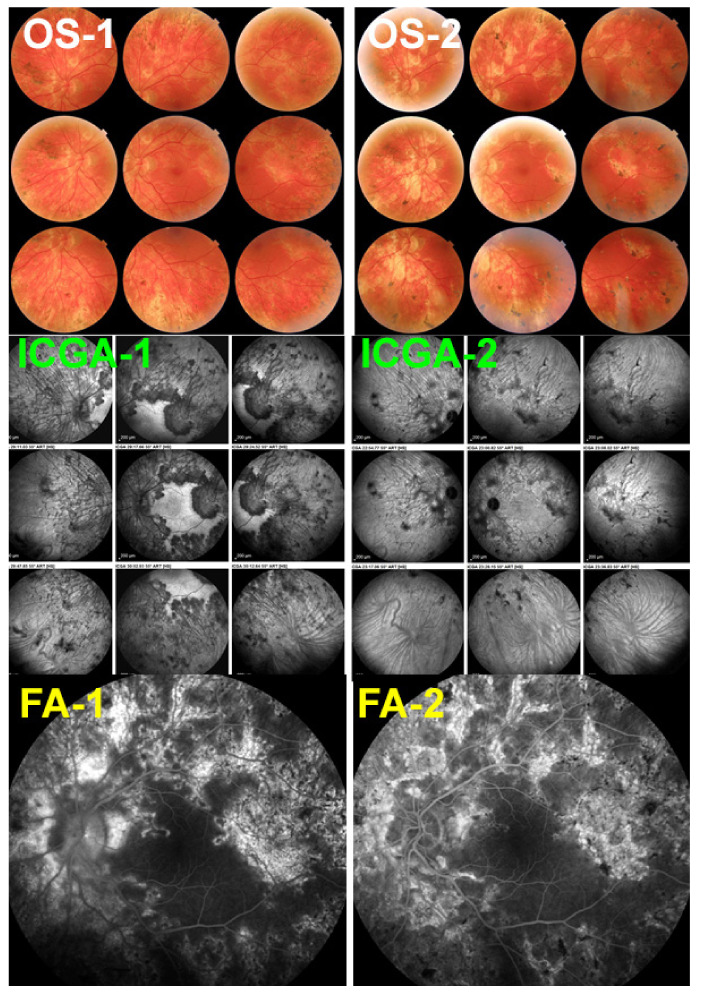
Tuberculosis related serpiginous choroiditis (TB-SC). Follow-up after treatment of the left eye (OS-1/OS-2; ICGA-1/ICGA-2; FA-1/FA-2) with fundus photography (**top images**) where changes apart from increased pigmentation are difficult to be identified. Similarly, FA (**bottom images**) is not very useful to monitor evolution after therapy. The most adequate modality to image improvement is ICGA (**middle images**).

Serpiginous-like choroiditis is a rare immune-mediated bilateral asymmetrically evolving sub-entity of tubercular uveitis affecting primarily the choriocapillaris and secondarily the retinal pigment epithelium (RPE), the outer retina and finally the whole chorio-retina (Figure 12). 

Gupta et al. first described this entity in seven patients in 2003 and reported a good response to antituberculosis therapy (ATT) [104]. The same group reported later that there was continuous progression of TB-SC after initiating ATT and they suggested increased immunosuppression [13].

Paradoxical worsening of TB-SC after introduction of ATT was reported, requiring increased immunosuppression [105]. Treatment is still controversial, and the discussion is whether initial treatment should consist of ATT alone or associated with immunosuppression. The Collaborative Ocular Tuberculosis Study Consensus Group has put forward complicated guidelines [106]. In our hands, as long as the IGRA test is positive, dual concomitant ATT (four drug scheme including isoniazid, rifampicin, ethambutol and pyrazinamide at onset of therapy and for 3 months) and multiple immunosuppressive therapy are needed to halt the progression of the disease [107]. The 3 months of 4-drug anti-TB treatment is followed by 2-drug therapy (isoniazid, rifampicin) for 7–9 months. Immunosuppressive therapy consists of oral corticosteroid 1 mg/kg/day, tacrolimus or CsA and azathioprine or MMF. and Biologic agents should be avoided or used with extreme caution as a reactivation of a latent tuberculosis may be lethal [108]. A particular case of very aggressive TB-SC uveitis was described by Tsui et al. Although aggressive immunosuppressive therapy and aggressive anti-tubercular therapy were introduced, the disease remained active. The patient was finally given 2 intravitreal methotrexate injections (400 μg/0.1 cc), at a one-month interval, that had stopped the evolution of the disease [56]. Adalimumab has been used safely after the completion of ATT in a patient with progressive TB-SC [109].

#### 5.1.6. Acute Syphilitic Posterior Placoid Chorioretinitis (ASPPC)

Acute syphilitic posterior placoid chorioretinitis (ASPPC) is a particular expression of syphilitic ocular involvement. The mechanism is a secondary choriocapillaritis with a presentation similar to APMPPE [14] (Figure 6). The fact that the process responds to corticosteroids despite the infectious nature of the trigger clearly points towards an immune-mediated mechanism [110]. Of course, the mainstay of therapy is specific antimicrobial treatment. ASPPC should be considered as neurosyphilis. Based on the CDC guidelines, current treatment with aqueous crystalline (benzyl) penicillin G 18–24 million units (MU) daily, administered as 3–4 million units intravenously every 4 hours or continuously infused for 10–14 days. Alternatively, 2.4 MU of IM procaine penicillin G once daily and probenecid 500 mg four times a day can be administered, both from 10 to 14 days [111]. It is very crucial to give an additional systemic corticosteroid therapy (0.5–0.8 mg/kg) at the start of therapy to avoid an ocular Jarisch-Herxheimer reaction [112].

### 5.2. Stromal Choroiditis

We include in this section only conditions for which the inflammatory process primarily starts in the choroidal stroma and not conditions that result from a systemic disease such as sarcoid chorioretinitis for which choroidal disease is just a chance random localization. We will not discuss either sympathetic ophthalmia, a disease with an identical pathophysiology to VKH disease for which the autoimmune reaction is triggered by a penetrating ocular injury.

#### 5.2.1. Vogt-Koyanagi-Harada Disease (VKH)

**Figure 13:**
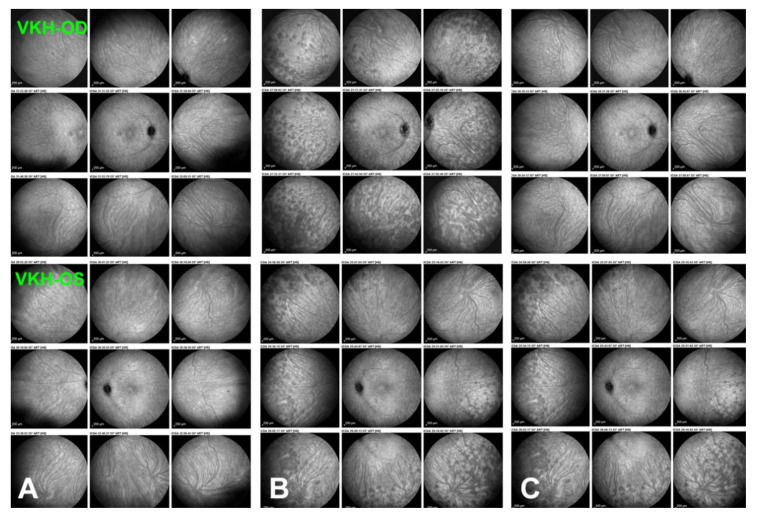
Monitoring of therapy using ICGA in a case of VKH. The situation of this patient is lesion free in both eyes under tapering prednisone, MMF and CsA (**A**). When prednisone was stopped completely extensive subclinical recurrence of HDDs (**B**), that responded to the administration of infliximab (5 mg/kg) (**C**). Subsequently the disease remained lesion-free during 6 years under the treatment of infliximab alone.

The specificity of VKH disease is the fact that the onset of the inflammatory autoimmune reaction starts exclusively in the choroidal stroma before the spill-over of inflammation involves adjacent ocular structures including the retina, vitreous, and optic disc, later further extending to the anterior segment [113] (Figure 13). Unlike other causes of chorioretinitis such as sarcoidosis which is a systemic disease that involves the choroid in a random chance fashion, in VKH the structure at the origin of inflammation, the choroidal stroma, is clearly identified and is influencing treatment strategies. Indeed, taking into account that inflammation develops in such a confined space, the effort will be oriented towards preventing the spread of inflammation to adjacent structures in order to avoid more global damage [114]. To achieve this goal, it became clear that early treatment was crucial to manage initial-onset acute disease [115]. The second important point to determine in order to achieve remission and even cure of disease, was the type of management required [116]. The time span within which remission or cure could be expected was estimated to be less than 3–4 weeks after onset of symptoms [117,118,119]. On the other hand, the type of therapeutical approach remained controversial up to recently [120,121]. One part of clinicians recommended corticosteroid monotherapy as the treatment of choice of initial-onset VKH [122]. More recently there are however more and more arguments in favour of first-line combined steroidal and non-steroidal immunosuppressive therapy [123,124,125]. A recent study based on a literature review unequivocally indicated that the dual first-line steroidal plus non-steroidal immunosuppressive therapy of initial-onset VKH disease was preferable to corticosteroid monotherapy [126]. In the latter study the outcomes of 892 patients (16 studies) having received corticosteroid monotherapy were compared to 172 patients (4 studies) having received first-line dual steroidal and non-steroidal immunosuppressive therapy. The proportion of patients who presented chronic evolution or development of sunset glow fundus was significantly lower in the latter dual treatment group than in the corticosteroid monotherapy group, being, respectively, 2.3% versus 44% for chronic evolution (*p* < 0.0001) and 17.5% versus 59% for sunset glow fundus (*p* < 0.0001). On the base of these results the dual steroidal and non-steroidal immunosuppression treatment approach appears clearly as the treatment of choice of initial-onset VKH. There is unanimous agreement on the use of initial systemic corticosteroid therapy. Although no long-term difference could be demonstrated when comparing high-dose (1000 mg) intravenous methylprednisolone for 3 days followed by high-dose oral prednisone versus high-dose oral prednisone (1.0–1.2 mg/kg) since onset of treatment [127], the first strategy is probably to be preferred, as rapid regression of inflammation should be sought in order to minimize tissue damage.

While the corticosteroid component of the dual treatment approach is generally accepted and well standardized, the choice of the additional nonsteroidal immunosuppressant is manifold. It appears that the decision to add a non-steroidal immunosuppressant at diagnosis is more important than the actual choice of the immunosuppressive agent. Table 3 is giving a list of the immunosuppressive agents used with success as first line complement to corticosteroid therapy in initial-onset disease in a literature search. It has to be noted that such studies are still rare.

Dual treatment has to be prolonged if successful discontinuation without disease recurrence, hence cure of the disease, is to be expected. In our hands, mean treatment duration of initial-onset VKH was 30.1 ± 34.6 months with no recurrence after treatment discontinuation [28] and in the study by Abu El-Asrar et al., immunosuppressive treatment was given for 20.1 ± 7.7 months [123]. This is much longer than what was usually recommended in the past. Our treatment protocol is, ideally, administration of high-dose corticosteroids, tapered to 0 after 6–8 months, combined with CsA (4.5 mg/kg per day) tapered to 0 after 9–12 months and Mycophenolic acid (1440 mg /day) maintained for at least 18–24 months. Evolution is checked for occult subclinical persistence or recurrence of lesions, using ICGA monitoring, followed by re-increase in dosages, if necessary. In case of insufficient response and/or unsatisfactory resolution of lesions, an anti-TNF-α agent is added. 

When the two prerequisites for successful management of initial-onset VKH disease, (1) early and (2) dual first-line immunosuppressive treatment associating steroidal and non-steroidal immunosuppressive therapy, are not fulfilled, there is a high probability for chronic evolution. It has been shown that high-dose corticosteroid therapy even when given very early in initial-onset disease is not preventing evolution towards chronicity and complications in a large proportion of cases [128]. On the other hand, when non-steroidal immunosuppression is not given concomitantly with corticosteroids, as first-line therapy but later, similarly, evolution towards chronicity and complications cannot be avoided [129], although some improvement has been noted [130].

The differentiation between two sub-entities of VKH, initial-onset versus chronic VKH has only recently been fully understood [116,119,131], as, indeed, response to treatment differs among these two sub-entities. As indicated earlier, the instauration of first line dual steroidal and non-steroidal immunosuppression is a recent practice with a high proportion of patients evolving towards chronicity when the additional immunosuppressant is added only later in the disease course.

Therefore, most articles on VKH therapy, concern chronically evolving patients. Treatment of chronic VKH is less successful, must be prolonged and usually needs a combination of several immunosuppressive agents usually determined empirically by trial and error.

Fortunately, for chronic VKH also, precise imaging investigations of the choroid such as ICGA, OCT and EDI-OCT [5,132,133] can be applied and are a precious help to rapidly determine whether introduced treatments are efficacious or not. Laser flare photometry (LFP) can measure the global level of intraocular inflammation and FA determines spill-over inflammation to the retina and represent additional monitoring devices. Using such investigational methods, it is nowadays possible to determine whether a given case responds or fails to respond to a chosen therapy allowing also to fine-tune treatment dosage [41]. Several studies were performed with additional non-steroidal Immunosuppressants added as second line therapy given more than 4 weeks after onset of symptoms and show some improvement over corticosteroid monotherapy but scarce cases of remission after discontinuation of therapy can be expected [134,135]. 

Numerous treatment options for chronic VKH have been published in series and case reports. Treatment in chronic VKH mostly needs to be continued for long periods to keep inflammation under control and treatment free follow-up is rare. 

A non-exhaustive literature review of case reports and case series of patients having benefited from additional immunomodulatory therapy in chronically evolving VKH disease was performed taking into account the last 15 years, a period during which most immunosuppressive agents used today became available. The search contained the terms VKH and immunosuppressive/immunomodulatory and biological treatments. We did not include in this search review articles or data from general articles on therapy of uveitis, but only reports directly dealing with treatment modalities for VKH disease and limited our search to the PubMed database.

Conventional immunosuppressants

**Figure 14:**
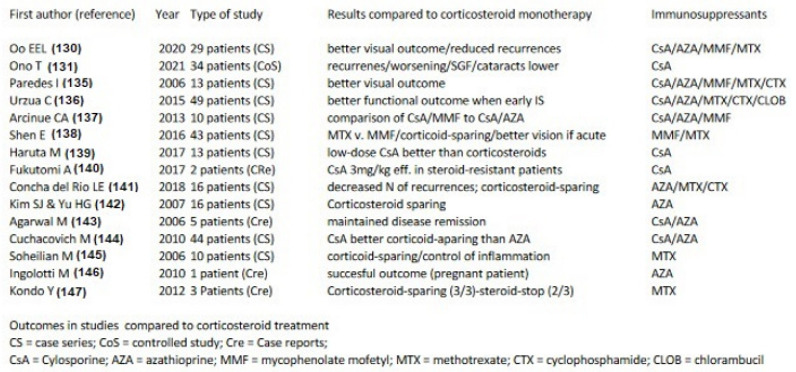
Studies on immunosuppressive agents used for chronic VKH disease.

The literature search on additional conventional immunosuppressive treatments in chronic VKH disease yielded 15 articles from 2006 till today [129,130,134,135,136,137,138,139,140,141,142,143,144,145,146] (Figure 14). The immunosuppressive agents reported include azathioprine (number of studies = 9), CsA (n = 9), MMF (n = 4), MTX (n = 7), cyclophosphamide (n = 2) and chlorambucil (n = 1). The number of immunosuppressive agents is higher than the number of studies as more than one agent was used in several studies. The beneficial effects listed were diverse and included mainly “control of inflammation” and “corticosteroid sparing effect”, but no case of remission after treatment discontinuation was reported.

Biologicals used to treat chronic VKH as a second line adjunct

**Figure 15:**
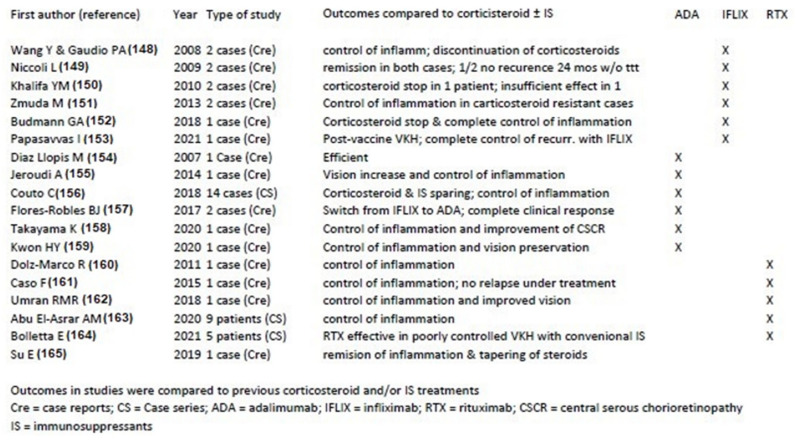
Studies on biological agents used for chronic VKH disease.

The literature search of studies on biological agents used for the treatment of chronic VKH yielded 18 articles from 2007 till today [147,148,149,150,151,152,153,154,155,156,157,158,159,160,161,162,163,164] (Figure 15), starting with the first article on the use of adalimumab for VKH [154]. The biological agents reported include adalimumab (number of studies = 7), infliximab (n = 6) and rituximab (n = 5). The beneficial effects listed were “control of inflammation” and remission. Among all the patients treated with biological agents, in a single patient only treated with infliximab no recurrence was reported after 24 months of follow-up without treatment [148]. The conclusions drawn from the articles on treatment with biologicals was the rapid and more effective response of VKH patients to such treatments when compared to conventional immunosuppressive therapies, which seems to make appear these agents as the treatment of choice to add to corticosteroid therapy as first-line treatment as well as second line adjunct treatment in chronic VKH patients.

In summary, the body of evidence available today is largely sufficient to indicate that first-line dual steroidal and non-steroidal immunosuppressive therapy within 3–4 weeks of initial-onset VKH disease is to be commended. It is the only strategy that allows us to reach not only remission but potential healing of disease altogether in a substantial proportion of cases [126]. Treatment has to be prolonged and subclinical monitoring by ICGA or EDI-OCT has to be used in order to detect subclinical inflammation in the choroid with consequent adaptation and fine-tuning of therapy [41]. Such close monitoring will also allow us to rapidly detect whether treatment options are effective or not in any given patient. If such a therapeutical protocol is not applied a large proportion of patients evolve to chronic disease for which healing is obtained only in rare cases. The therapy needed is that of multiple immunosuppressants for prolonged periods with numerous relapses and complications. For chronic VKH disease, numerous conventional immunosuppressive and/or biological therapies have been used in addition of corticosteroids showing benefits on inflammation (Figure 14 and Figure 15). Biologicals seem to yield better effects when compared to conventional immunosuppressants.

#### 5.2.2. HLA-A29 Birdshot Retinochoroiditis (BRC)

**Figure 16:**
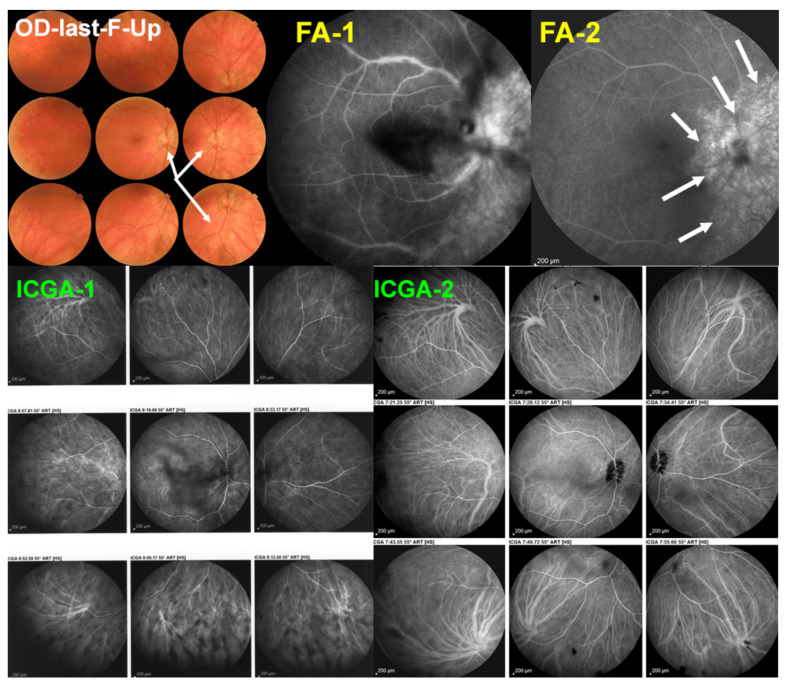
Monitoring HLA-A29 BRC using fundus photography, FA and ICGA: case of HLA-A29-BRC treated early with multiple immunosuppressants. After more than 8 years of treatment typical “birdshot lesions” never developed (**top left image**), although there is some pallor peripapillary and infero-nasally to the disc (white arrows) explained by the retinal involvement. Monitoring of retinal disease is necessary and performed by FA. At presentation (**FA-1**) substantial retinal vasculitis is present which has resolved at last follow-up (**FA-2**) leaving peripapillary atrophy (hyperfluorescent area (white arrows). Monitoring of choroidal disease is achieved through ICGA that shows many HDDs and unrecognizable choroidal vessels at presentation (**ICGA-1**) having completely responded to therapy after more than 8 years of follow-up (**ICGA-2**).

HLA-A29 birdshot retinochoroiditis (BRC) is a presumed autoimmune disease possibly targeting stromal melanocytes and is classified as a stromal choroiditis [165]. Unlike for VKH, the molecular target of the immune reaction is not known, and the inflammatory reaction does not involve the whole choroidal thickness but is limited to non-confluent smaller multiple inflammatory foci that remain confined to the mid-stroma [166] with limited or without spill-over to adjacent structures (Figure 16). The fundamental difference between BRC and VKH is the concomitant parallel retinal involvement unrelated to the choroidal inflammation occurring in BRC (Figure 16) [167] Morbidity in BRC is mainly caused by the retinal and vitreous inflammation [34] and not by the choroiditis which is however causing choroidal thinning in the long run [36]. In active disease, the choroiditis is characterized by the ICGA HDDs that give precious indications on the level of disease activity. ICGA is a useful biomarker, crucial to determine disease activity but also to monitor response to treatment, allowing to assess rapidly whether an agent is efficient or not in a given patient [168,169]. The knowledge of the pathophysiology of BRC, dual choroidal and retinal inflammation, determines the therapeutical approach of the disease. Indeed, the impact of immunosuppression on vitreous and retinal inflammation is attenuated because agents have to cross the blood-ocular barriers and dosages have to be adapted accordingly. 

Several treatment principles for BRC have emerged in recent years. (1) In the past BRC was considered as an indolent slowly progressing disease that tended to stabilize and maintain a relatively good visual acuity not needing systemic therapy [170,171,172,173]. Contrary to what was thought to be the case then, when the disease was first diagnosed, some forty years ago, early and vigorous immunosuppressive therapy rather than observation without treatment, is the management of choice for BRC. Indeed, up to 85% of cases have a deleterious evolution with retinal and choroidal atrophy if no immunosuppressive therapy is administered [174]. (2) Treatment should not only be vigorous but should also be initiated early after diagnosis. The use of ICGA has made it possible to diagnose BRC before the hallmark oval rice-shaped depigmented fundus “birdshot lesions” become apparent, (Figure 1) by showing subclinical HDDs [175]. Such an early diagnosis allowed us to start treatment at an initial stage of disease which prevented the development altogether of the characteristic rice-shaped depigmented BRC fundus lesions [176]. (3) Prolonged treatment is usually necessary, as discontinuation of treatment is often followed by recurrences [177]. Therefore, well-tolerated immunosuppressive therapy should be sought and corticosteroid therapy should be tapered as early as possible, used only to curb inflammation until corticosteroid sparing immunosuppressive agents are fully active. (4) Unlike in VKH, where local treatments are inappropriate, as the inflammatory process is exclusively developing from the choroid, local peri-ocular or intra-ocular corticosteroid injections for BRC can sometimes be used as an adjuvant therapy particularly in two situations: (a) in case of initial-onset unilateral disease, periocular sub-Tenon’s triamcinolone injections (40 mg) can delay the use of systemic immunosuppressive treatment for some time [174]. (b) Another situation that can justify the use of intraocular corticosteroid releasing devices, is when aggressive systemic immunosuppression fails to sufficiently control retinal disease.

Our treatment protocol is ideally including administration of oral prednisone (0.8–1 mg/kg) at the start of treatment, tapered to 0 after 6–8 months, associated with a quickly acting immunosuppressant (CsA (4.5 mg/kg per day) or tacrolimus (0.05–0.15 mg/kg)) tapered to 0 after 9–12 months and further associated with Mycophenolic acid (1440 mg /day) maintained for several years. The length of treatment is not established with one study reporting the treatment duration of 30 months to induce a substantial percentage of remissions without treatment [178]. In our hands, we were unable to discontinue treatment without recurrences in almost all our patients followed for more than 10 years [36]. Evolution is checked for occult subclinical persistence or recurrence of lesions, using ICGA monitoring, followed by re-increase in dosages, if necessary. In case of insufficient response and/or unsatisfactory resolution of lesions, a biological immunomodulator, usually an anti-TNF-α agent, is added. As indicated earlier the presented protocol can be delayed in case of mild initial predominantly unilateral involvement in which case sub-Tenon’s injections of triamcinolone before systemic therapy are applied [174].

**Figure 17:**
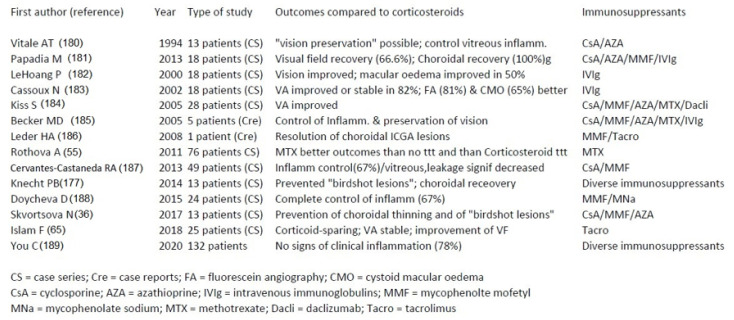
Studies on Immunosuppressive agents used for HLA-A29-BRC.

Conventional immunosuppressants used for HLA-A-29-BRC ( Figure 17 )

The different agents used successfully in BRC have been listed following a non-exhaustive literature search limited to the PubMed database (Figure 17). The search contained the terms birdshot retinochoroiditis/chorioretinitis and immunosuppressive / immunomodulatory treatments or biological treatments. We carried out not include in this search review articles or data from general articles on therapy of uveitis, but only reports directly dealing with treatment modalities for HLA-A29 BRC. 

Fourteen articles on the use of conventional immunosuppressive therapies, mostly in addition to systemic corticosteroids, were identified [36,55,65,176,179,180,181,182,183,184,185,186,187,188] (Figure 17). Six articles used mycophenolate mofetil, five used cyclosporine, 3, respectively, used azathioprine, methotrexate and intravenous immunoglobulins, two used tacrolimus and in two studies corticosteroids alone were reported as efficient (not reported here).

Biologicals used for HLA-A29-BRC

Proportionally, studies on the use of biologicals for BRC (n = 7) were less frequent than for VKH disease (n = 18). In 2008, Sobrin et al. used daclizumab, a humanised monoclonal antibody that blocks interleukin-2 receptor, inhibiting effector T cell expansion, in 8 patients [189]. In 7/8 patients either stabilisation or improvement of visual acuity was obtained. Six patients had resolution of vasculitis on FA and four patients were able to discontinue all other immunosuppressive treatments. Two studies showed a positive effect of adalimumab in 22 patients [190,191]. Beneficial effects included improvement of visual acuity, discontinuation of conventional immunosuppressants and decrease in cystoid macular oedema (CMO). However, one study showed that adalimumab monotherapy did not prevent the recurrence of HDDs on ICGA in two patients [192]. Two studies reported on four patients resistant to multiple immunosuppressants and biologicals, who responded to tocilizumab by improving visual acuity, by having a corticosteroid sparing effect and producing a decrease in CMO [74,193]. Infliximab was used in 22 patients, refractory to conventional immunosuppressive agents and other biologicals, being effective to control inflammation in otherwise treatment-refractory cases of HLA-A29-BRC [194]. Control of inflammation was achieved in 88.9% of patients, improved visual acuity was found in 94.4% of eyes and the proportion of patients with CMO was reduced by two thirds.

Local, periocular and intraocular therapies for HLA-A29-BRC

Thirteen articles on the use of local periocular or intraocular corticosteroid treatments for BRC were identified in the literature search whereas this treatment modality is rarely used for VKH disease, as the inflammation focus is in the choroid and, therefore, more easily accessible by systemic therapy through its very strongly vascularized net. In 5 reports fluocinolone acetonide intravitreal implants were used [195,196,197,198,199], in 4 dexamethasone intravitreal implants [200,201,202,203], and in another 4 intravitreal or sub-Tenon’s triamcinolone acetonide injections [174,204,205,206].

As far as the use of the fluocinolone acetonide implant is concerned, no reasons were given for its use in two reports and refractoriness to systemic immunosuppression in another two, especially non-response of retinal inflammation. Indeed, the treatment was beneficial on retinal inflammatory signs such as vasculitis and CMO. In all studies other systemic immunosuppressive treatments could be significantly decreased or stopped. The drawback was the relatively high proportion of ocular side-effects including glaucoma and development of cataract. A few patients have been treated with the dexamethasone implant with transient good effects on retinal inflammatory parameters and delay of introduction of systemic immunosuppression. Intraocular or periocular triamcinolone was mainly useful to avoid the use of systemic immunosuppression in patients with a benign evolution or to delay the initiation of systemic immunosuppressive therapy.

Interestingly, two reports showed that intraocular administration of corticosteroids had no effect on choroidal disease, as can be expected from the pharmacokinetics of this type of therapeutical approach [192,199].

Stromal choroiditis in BRC is moderate and easy to treat, whereas the therapeutic problem comes from the independent but concomitantly occurring retinal involvement which is causing most of the disease morbidity. As for VKH disease, non-steroidal immunosuppression is unavoidable in BRC as shown by the 14 studies using conventional immunosuppressants or the seven studies using biological agents found in the literature search. Unlike for VKH disease, there are fewer studies on the use of biological agents.

However, reports on the use of intraocular or periocular use of corticosteroids are numerous, indicating that management of the retinal involvement is the difficult component to treat which can profit from such local treatments, that are absolutely inappropriate for VKH disease.

## 6. Conclusions

Non-infectious choroiditis could be approached in a precise way only once the means of investigation of this structure were available. Prior to this, limited imaging access has led to speculations about mechanisms involved, that generated erroneous interpretations and classifications such as the inappropriate terminology of “white dot syndromes”.

Since ICGA became available, it has become possible to apply pioneering pragmatism allowing to understand the pathophysiology of non-infectious choroiditis and, hence, made it possible to classify these conditions and above all to monitor their evolution, check the efficacy and response to treatments. Other imaging techniques have now been added to ICGA which, nevertheless, remains the most useful technique for non-infectious choroiditis. Thanks to this improved imaging access to the choroid showing occult subclinical inflammatory involvement, it became obvious that most of these conditions needed heavy dual and even triple immunosuppression and the use of biologic agents in refractory cases.

## Figures and Tables

**Table 1:** Antimetabolites main features.

	Mechanism of Action	Dose	Side Effects
**Azathioprine**	Purine analogue which interferes with DNA and RNA synthesis. Cytostatic drug for T-cells	2.25–2.75 mg/kg/day (not if absence of TPMT)	bone marrow suppression hepatic toxicity upper gastrointestinal symptoms
**Mycophenolate Mofetil (MMF)/Mycophenolic Acid (MA)**	Inhibit the inosine-5′monophosphate dehydrogenase ⇒ stop the purine biosynthetic pathways/decrease antibody production of B-cells. Strong cytostatic effect on T and B lymphocytes	MMF: 1–3 g/day MA: 1440 mg/day	gastrointestinal discomfort bone marrow depression hepatic disfunction not compatible with pregnancy
**Methotrexate**	Inhibits dihydrofolate reductase ⇒ reduction in DNA and RNA synthesis of rapidly dividing cells	7.5 to 25 mg/week	hepatic toxicity gastrointestinal upset leukopenia (bone marrow suppression)
**Calcineurin Inhibitors (CsA/Tarcolimus)**	Block T lymphocytes by suppressing the production of IL-2, a major enhancer for T-cell activation and recruitment	CsA: 3–5 mg/kg/day Tarcolimus:0.05–0.15 mg/kg/day	renal toxicity arterial hypertension dyslipidaemia hirsutism

DNA: Deoxyribonucleic acid, RNA: Ribonucleic acid, TPMT: thiopurine S-methyltransferase, mg: milligrams, kg: kilogrammes, g: grams, CsA: Cyclosporine.

**Table 2:** Biological Agents main features.

	Mechanism of Action	Dose	Side Effects
**Infliximab [70]**	Chimeric monoclonal antibody, bound to both transmembrane and soluble form of TNF-a. Kills cells that express TNF-a	iv 5–20 mg/kg/day Loading dose at 0, 2, 4 weeks then every 6–10 weeks	Reactivation of infections Lupus-like syndrome Malignancy (lymphoproliferative disease)
**Adalimumab [76,77]**	Human monoclonal antibody, same as infliximab	SC 40 mg every 2 weeks (in severe cases interval can decrease to 7–10 days [3])	Headache, nausea, stomachache Secondary malignancy Demyelinating disorder
**Rituximab [78]**	Chimeric anti-CD20 monoclonal antibody, targets peripheral CD20 B-cells	iv 375 mg/m^2^ every week for 8 w then every 4 w for 4 months	Infusion reactions Neutropenia Progressive multifocal leukoencephalopathy
**Anakinra [79]**	Humanized monoclonal IgG antibody, anti-IL-1 receptor	100 mg/day	Infusion site infection Upper respiratory infections Neutropenia
**Tocilizumab [80]**	Humanized monoclonal antibody, anti-IL 6 receptor	8 mg/kg every 4 w	Serious infections Neutropenia Allergy/anaphylaxis

TNF: tumor necrosis factor; IL: interleukin; IV: intravenous / SC: subcutaneous; mg: milligram /kg: kilogram; w: weeks.

**Table 3:** Studies reporting on results of dual first-line steroidal and non-steroidal treatment in patients presenting with initial-onset Vogt-Koyanagi-Harada disease.

Author	Year	N of Patients	Treatment	Number of Patients with Chronicity (%)	Number of Patients with SGF (%)
Bouchenaki [28]	2011	5	CS + IST	0 (0)	0 (0)
Abu El Asrar [123]	2017	38	CS + MMF	0 (0)	0 (0)
Lodhi [125]	2017	24	CS + AZA	4 (17)	6 (25)
Yang [118]	2018	105	CS + IST	0 (0)	24 (23)
Total		172	CS + IST	4/172 = 2.3%	30/172 = 17.5%

SGF: sunset glow fundus; CS: corticosteroid; IST: diverse conventional and validated immunosuppressive treatments; MMF: Mycophenolate Mofetil; AZA: azathioprine.

## Data Availability

Data sharing not applicable.
